# Maternal smoking during pregnancy negatively affects brain volumes proportional to intracranial volume in adolescents born very preterm

**DOI:** 10.3389/fnhum.2022.1085986

**Published:** 2023-01-05

**Authors:** Mikael O. Ekblad, Peter Ngum, Harri Merisaari, Virva Saunavaara, Riitta Parkkola, Sirkku Setänen

**Affiliations:** ^1^Department of General Practice, Institute of Clinical Medicine, University of Turku and Turku University Hospital, Turku, Finland; ^2^Turku Brain Injury Center, Turku University Hospital and University of Turku, Turku, Finland; ^3^Department of Radiology, University of Turku, Turku, Finland; ^4^Turku Brain and Mind Center, University of Turku, Turku, Finland; ^5^Division of Medical Imaging, Department of Medical Physics, Turku University Hospital, Turku, Finland; ^6^Turku PET Centre, University of Turku and Turku University Hospital, Turku, Finland; ^7^Department of Radiology, Turku University Hospital, Turku, Finland; ^8^Department of Pediatric Neurology, University of Turku and Turku University Hospital, Turku, Finland

**Keywords:** cigarette, FreeSurfer, exposure, long-term, MRI, nicotine, prenatal, ROI

## Abstract

**Background:**

Maternal smoking during pregnancy has been shown to associate with smaller frontal lobe and cerebellar volumes in brain magnetic resonance imaging (MRI) at term age in very preterm infants. The aim of this study was to examine the effect of maternal smoking during pregnancy on volumetric brain MRI findings at 13 years. We hypothesized that adverse effects of smoking during pregnancy on brain volumes are still seen during adolescence.

**Methods:**

Included adolescents were born very preterm (gestational age < 32 weeks and/or birth weight ≤ 1,500 g) between April 2004 and December 2006 at the Turku University Hospital, Finland. Information on maternal smoking status (yes or no) during pregnancy was collected from medical records and maternal questionnaires before discharge. Adolescents underwent volumetric brain MRI at 13 years of age. Image post-processing was performed with FreeSurfer. Regional volumes, cortical thickness, surface area, and curvature were computed from 33 cortical regions of interest (ROIs). Additionally, volumes were calculated for 18 subcortical regions, as well as for white matter, gray matter, and intracranial volume. We normalized quantified absolute volumes for head size by dividing volumes with corresponding intracranial volumes. false discovery rate (FDR) correction for multiple comparisons across regions was used.

**Results:**

A total of 9/44 (21%) adolescents had been exposed to maternal smoking during pregnancy. No statistically significant differences in absolute volumes were observed between the groups (*p* > 0.05). Regarding volumes proportional to intracranial volume, the adolescents in the exposed group exhibited smaller gray matter volumes in the inferotemporal (FDR corrected *p* = 0.022) and parahippocampal (*p* = 0.018) regions compared to the unexposed group. The surface area in the exposed group was also smaller in the parahippocampal (*p* = 0.046) and postcentral (*p* = 0.046) regions compared to the unexposed group. No statistically significant differences after correction for multiple comparisons were found for either curvature or cortical thickness between the groups.

**Conclusion:**

Maternal smoking exposure during pregnancy may have long-term effects on brain volumes up to 13 years in adolescents born very preterm. Our findings emphasize the importance of smoking-free pregnancy.

## 1. Introduction

Smoking during pregnancy is known to increase the risk of low birth weight, smaller head circumference, and preterm birth (Ekblad et al., 2015; Ion and Bernal, 2015; Abraham et al., 2017). The adverse effects of smoking would be completely preventable by smoking cessation prior to pregnancy. Although the harms of smoking are widely known, on average, 6–10% of pregnant women still smoke in the American and European regions (Lange et al., 2018). In Finland, 18% of very preterm infants born in 2001–2006 were exposed to maternal smoking during pregnancy (Ekblad et al., 2010).

The most significant harmful substances in tobacco are nicotine and carbon monoxide, which cross the placenta and enter the fetal circulation affecting fetal development (Ekblad et al., 2015). Nicotine binds to the nicotinic acetylcholine receptors, which are already present in the fetal brain and spinal cord from 4–5 weeks of gestation (Hellström-Lindahl et al., 1998). During fetal brain development, the nicotinic acetylcholine receptors modulate axonal pathfinding, synapse formation, and cell survival (Role and Berg, 1996; Ekblad et al., 2015). It has been shown that smoking exposure during pregnancy affected DNA methylation of the developing dorsolateral prefrontal cortex during the second trimester of gestation among 24 fetuses aborted for non-medical reasons (Chatterton et al., 2017). Smoking exposure was also associated with reduced mature neuronal content in the dorsolateral prefrontal cortex, speculated to be driven by nicotine in tobacco.

Functional magnetic resonance imaging (MRI) studies have demonstrated that smoking exposure during pregnancy leads to long-term functional brain changes that persist into adolescence (Bublitz and Stroud, 2012). Previous literature has shown long-lasting adverse effects of smoking exposure during pregnancy on brain morphology, e.g., smaller total brain volumes, compared to unexposed (El Marroun et al., 2014; Zou et al., 2022). A recent prospective population-based cohort study investigated the association between maternal smoking during pregnancy and volumetric brain MRI findings in 10-year-old children born full-term (Zou et al., 2022). Exposure to smoking throughout pregnancy, but not exposure during early pregnancy only, was associated with lower total brain volume, lower cerebral gray matter volume, lower cerebral white matter volume, smaller surface area, and less gyrification compared to exposure. In very preterm infants, we have previously shown that maternal smoking exposure during pregnancy was associated with smaller frontal lobe and cerebellar volumes but not with total brain volumes in brain MRI at term (Ekblad et al., 2010). However, the total brain volume or head growth during the first 2 years of life did not differ according to maternal smoking status during pregnancy (Ekblad et al., 2010).

In addition to brain development, maternal smoking is known to affect the neurodevelopment of the child (Bublitz and Stroud, 2012; Ekblad et al., 2015; Froggatt et al., 2020). Very preterm birth also increases the risk for neurodevelopmental impairments (Setänen et al., 2013). According to a recent systematic review and meta-analysis, smoking during pregnancy affected neurobehavioral functioning during the first year of life in the general pediatric population (Froggatt et al., 2020). Additionally, extremely preterm birth has been shown to affect neurobehavioral functioning from childhood to early adulthood (Linsell et al., 2019). An important gap in the current literature is that there are no studies on the long-term effects of smoking during pregnancy on brain volumes in adolescents born very preterm.

The present prospective study sought to examine the effect of maternal smoking during pregnancy on volumetric brain MRI findings at 13 years in a cohort of adolescents born very preterm in the 2000s. We hypothesized that adverse effects of smoking during pregnancy on brain volumes would still be seen at 13 years.

## 2. Materials and methods

**FIGURE 1:**
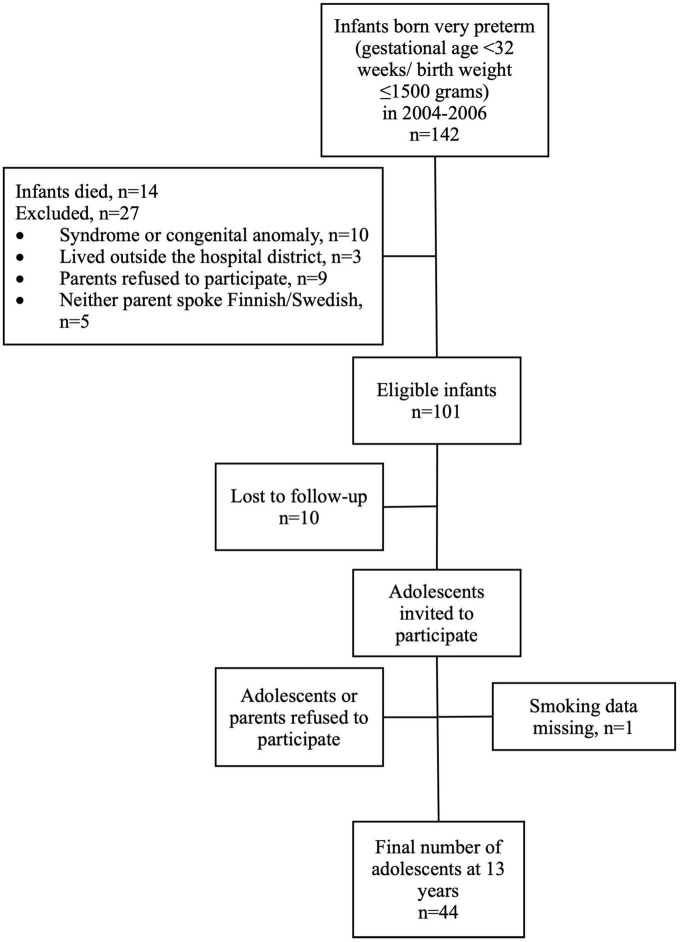
Flow chart of the study participants.

This study is part of the prospective PIPARI Study (The development and functioning of very low birth weight infants from infancy to school age) of very preterm infants. The participants in the present study were born to Finnish or Swedish speaking families from April 2004 to December 2006 in Turku University Hospital, Finland. The inclusion criteria were gestational age < 32 weeks and/or birth weight ≤ 1,500 g. The exclusion criteria were severe congenital anomalies or diagnosed syndrome affecting development. Only adolescents with information on maternal smoking status during pregnancy were included. The flow chart of the participants is shown in Figure 1.

Perinatal characteristics (Table 1) were obtained from the medical records using the Vermont Oxford Network criteria. Information on maternal smoking status (yes or no) during pregnancy was collected from medical records and maternal questionnaires before discharge, as described previously (Ekblad et al., 2010).

**TABLE 1:** Perinatal background characteristics of all study adolescents born very preterm (gestational age < 32 weeks and/or birth weight ≤ 1,500 g), and comparison between those exposed and unexposed to maternal smoking during pregnancy (*p*-value).

	All study adolescents, *n* = 44	Exposed group, *n* = 9	Unexposed group, *n* = 35	*P*-value
Gestational age, mean (SD), week	28.9 (2.8)	30.4 (1.9)	28.6 (2.8)	0.08
Birth weight, mean (SD), grams	1,174.6 (382.6)	1,302.2 (408.1)	1,141.7 (374.9)	0.3
Small for gestational age (<−2 SD) [*n* (%)]	58 (33)	3 (33.3)	12 (34.3)	1.0
Birth weight *z*-score, mean (SD)	−1.2 (1.6)	−1.6 (1.5)	−1.1 (1.6)	0.4
Prenatal corticosteroids [*n* (%)]	43 (97.7)	9 (100.0)	34 (97.1)	1.0
Male [*n* (%)]	28 (63.6)	9 (100.0)	19 (54.3)	0.02
Cesarean delivery [*n* (%)]	24 (54.5)	4 (44.4)	20 (57.1)	0.7
Multiple birth [*n* (%)]	13 (29.5)	4 (44.4)	9 (25.7)	0.4
Bronchopulmonary dysplasia [*n* (%)]	6 (13.6)	1 (11.1)	5 (14.3)	1.0
Operated necrotizing enterocolitis [*n* (%)]	1/41 (2.4)	0/9 (0.0)	1/32 (3.1)	1.0
Sepsis [*n* (%)]	2 (4.5)	1 (11.1)	1 (2.9)	0.4
Laser-treated retinopathy of prematurity [*n* (%)]	2/41 (4.9)	0/9 (0.0)	2/32 (6.3)	1.0
Major brain pathologies at term* [*n* (%)]	12/42 (28.6)	1/9 (11.1)	11/33 (33.3)	0.2
Mother’s education > 12 years [*n* (%)]	14/37 (37.8)	1/7 (14.3)	13/30 (43.3)	0.2
Father’s education > 12 years [*n* (%)]	7/37 (18.9)	0/7 (0.0)	7/30 (23.3)	0.3
Cerebral palsy [*n* (%)]	2 (4.5)	0 (0.0)	2 (5.7)	1.0
Full-scale intelligence quotient < 70 at 11 years [*n* (%)]	7/40 (17.5)	3/9 (33.3)	4/31 (12.9)	0.3

*The specific magnetic resonance imaging (MRI) protocol and details about the classification of the findings have been previously described by Setänen et al. (2013).

### 2.1. Image acquisition

Each MRI examination, performed on 3T Philips Ingenuity TF PET/MR (Philips, Amsterdam, Netherlands), started with structural scans: a 3D T1-weighted turbo-field-echo scan in sagittal orientation with 8.1 ms repetition time (TR), 3.7 ms echo time (TE), and isotropic 1 mm voxel; a T2- weighted turbo-spin-echo scan in axial orientation with 3,756 ms TR, 80 ms TE, 3 mm slice thickness with 0.5 mm gap between slices. Images were reconstructed with 0.45 mm × 0.45 mm pixel size; a coronal fluid attenuation inversion recovery scan in coronal orientation with 10, 000 ms TR, 2,800 ms inversion time, TE of 125 ms, slice thickness of 4 mm, and the gap between slices 1 mm. Images were reconstructed with 0.45 mm × 0.45 mm pixel size.

### 2.2. Image analysis

FreeSurfer version 7.2.0 was used for cortical reconstruction and volumetric segmentation of T1-weighted images (Dale et al., 1999; Fischl et al., 2002). Segmentations were visually inspected. Regional volume, cortical thickness, surface area, and curvature were computed from 33 cortical regions of interest (ROIs). Additionally, volumes were calculated for 18 subcortical regions, as well as for white matter, gray matter, and intracranial volume. The ROIs are presented in Supplementary Table 1.

### 2.3. Statistical analysis

The normality of the distributions was assessed both graphically and with the Shapiro–Wilk test. Continuous variables were described by means (SD) or medians (interquartile range) depending on their distribution. Differences in continuous background characteristics between the groups (maternal smoking status during pregnancy or study participants and drop-outs) were studied using the independent sample *T*-test. For the categorical background characteristics, chi-square test or Fisher’s exact test was used. The statistical analyses were performed using an SPSS version 28.0 (IBM SPSS Statistics, IBM Corporation, NY, USA). A two-tailed *p*-value of < 0.05 was considered statistically significant.

For volumetric analyses, quantitative measures were used for group comparisons of the 33 cortical regions, 18 subcortical regions, white matter, gray matter, and intracranial volume using Wilcoxon rank sum test. In addition, we applied regression analysis, depicted in Table 2, to assess the association between quantified volumes and the mother’s smoking status. *P*-values *p* < 0.05 after false discovery rate (FDR) correction for multiple comparisons across regions were considered statistically significant. The statistical analyses for MRI derivatives were performed with R (version 4.1.2). FDR corrected values are reported unless otherwise noted. We normalized quantified absolute volumes for head size by dividing volumes with corresponding intracranial volumes (ICV).

**TABLE 2:** Regression analysis models for adolescents born very preterm (gestational age < 32 weeks and/or birth weight ≤ 1,500 g).

Model type	Model formula
Logistic regression	Status ∼ T1W + birth weight *z*-score + age_mother_
Logistic regression	Status ∼ T1W + sex + gestational age (days)
Regression	T1W ∼ status + sex + gestational age (days)

Maternal smoking exposure during pregnancy (yes/no). T1W: one of: cortical region of interest (ROI) volume (ml), cortex thickness (mm), and ROI cortex area. age_mother_, mother’s age in years; birth weight *z*-score.

## 3. Results

A total of 44 adolescents born very preterm underwent volumetric brain MRI at mean age of 12.8 years (SD = 0.5). A total of nine (21%) of them had been exposed to maternal smoking during pregnancy. Perinatal background characteristics are shown in Table 1. All adolescents in the exposed group were boys (100% vs. 54% in the unexposed group, *p* = 0.02). Study participants had lower gestational age (mean 28.9 weeks vs. 30.2 weeks) and lower birth weight (1,174.6 g vs. 1,307.1 g) than drop-outs. There were no other differences in perinatal background characteristics or maternal smoking exposure during pregnancy between study participants and drop-outs.

In the exposed group, the median (interquartile range) absolute volumes for white matter, gray matter, and intracranial volume were 462.4 (27.2), 753.1 (38.6), and 1,553 (126.7) ml. For the unexposed group, the corresponding volumes were 406.5 (93.8), 708 (120.9), and 1,406.5 (198.8) ml. No statistically significant differences in absolute volumes were observed between the groups (*p* > 0.05). In the regression analysis, the status of the exposure group did not contribute statistically significantly to volumetric data, and in logistic regression analysis, the volumetric data did not contribute significantly to differentiation between the groups.

**FIGURE 2:**
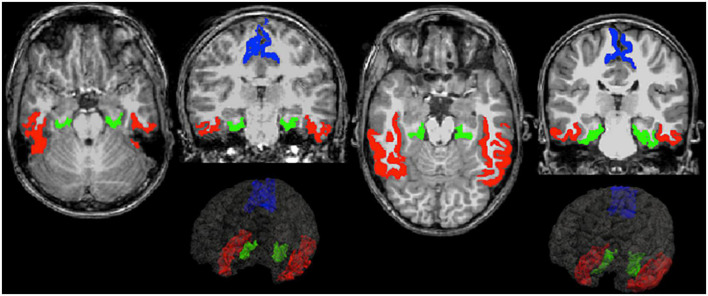
Example figures of inferior temporal (red), parahippocampal (green), and postcentral (blue) regions where differences were found between exposed **(left)** and unexposed **(right)** group of adolescents born very preterm in transaxial and coronal slices of T1-weighted magnetic resonance imaging (MRI) images.

Regarding volumes proportional to intracranial volume, the adolescents in the exposed group exhibited smaller gray matter volumes in the inferotemporal (*p* = 0.002, FDR corrected *p* = 0.022) and parahippocampal (*p* < 0.001, FDR corrected *p* = 0.018) regions compared to the unexposed group (Figure 2 and Table 3). The surface area in the exposed group was also smaller in the parahippocampal (*p* < 0.001, FDR corrected *p* = 0.046) and postcentral (*p* = 0.004, FDR corrected *p* = 0.046) regions compared to unexposed group. No statistically significant differences after correction for multiple comparisons were found for either curvature or cortical thickness between the groups (Figure 2 and Table 3), nor volumes of subcortical regions (Supplementary Table 2).

**TABLE 3:** Differences in gray matter volume, cortical thickness, surface area, and mean curvature between adolescents born very preterm exposed and unexposed to maternal smoking during pregnancy.

	Gray matter		Cortical thickness		Surface area		Mean curvature	
**ROI**	***P*-value**	**FDR *P*-value**	***P*-value**	**FDR *P*-value**	***P*-value**	**FDR *P*-value**	***P*-value**	**FDR *P*-value**
Caudal anterior cingulate	0.254	0.725	0.124	0.535	0.188	0.762	0.177	0.955
Caudal middle frontal	0.977	1.000	0.977	0.977	0.967	0.986	0.501	0.955
Cuneus	0.524	0.797	0.644	0.924	0.465	0.790	0.279	0.955
Entorhinal	0.323	0.761	0.067	0.369	0.329	0.769	0.300	0.955
Fusiform	0.264	0.725	0.644	0.924	0.192	0.762	0.874	0.959
Inferior parietal	0.774	0.852	0.636	0.924	0.757	0.960	0.636	0.955
**Inferior temporal**	0.001	**0.022**	0.037	0.369	0.005	0.056	0.539	0.955
Isthmus cingulate	0.235	0.725	0.893	0.959	0.254	0.762	0.874	0.959
Lateral occipital	0.721	0.852	0.244	0.672	0.856	0.986	0.792	0.955
Lateral orbitofrontal	0.570	0.806	0.884	0.959	0.396	0.769	0.695	0.955
Lingual	0.139	0.654	0.180	0.564	0.192	0.762	0.026	0.852
Medial orbitofrontal	0.586	0.806	0.930	0.959	0.509	0.799	0.603	0.955
Middle temporal	0.383	0.791	0.686	0.944	0.479	0.790	0.774	0.955
**Parahippocampal**	0.001	**0.018**	0.009	0.147	0.003	**0.046**	0.173	0.955
Paracentral	0.757	0.852	0.430	0.886	0.986	0.986	0.184	0.955
Pars opercularis	0.377	0.791	0.340	0.802	0.377	0.769	0.352	0.955
Pars orbitalis	0.416	0.797	0.531	0.924	0.555	0.832	0.669	0.955
Pars triangularis	0.730	0.852	0.930	0.959	0.619	0.851	0.235	0.955
Peri-calcarine	0.016	0.129	0.064	0.369	0.022	0.183	0.739	0.955
**Postcentral**	0.005	0.060	0.008	0.147	0.003	**0.046**	0.594	0.955
Posterior cingulate	0.230	0.725	0.188	0.564	0.235	0.762	0.479	0.955
Precentral	0.619	0.817	0.365	0.802	0.371	0.769	0.686	0.955
Precuneus	0.121	0.654	0.067	0.369	0.221	0.762	0.911	0.959
Rostral anterior cingulate	0.531	0.797	0.721	0.952	0.465	0.790	0.089	0.955
Rostral middle frontal	0.531	0.797	0.765	0.959	0.704	0.929	0.810	0.955
Superior frontal	0.323	0.761	0.594	0.924	0.284	0.769	0.611	0.955
Superior parietal	1.000	1.000	0.555	0.924	0.986	0.986	0.930	0.959
Superior temporal	0.244	0.725	0.180	0.564	0.390	0.769	0.695	0.955
Supramarginal	0.103	0.654	0.130	0.535	0.155	0.762	0.184	0.955
Frontal pole	0.479	0.797	0.340	0.802	0.977	0.986	0.274	0.955
Temporal pole	0.531	0.797	0.874	0.959	0.619	0.851	0.611	0.955
Transverse temporal	0.704	0.852	0.486	0.924	0.958	0.986	0.995	0.995
Insula	0.967	1.000	0.865	0.959	0.939	0.986	0.757	0.955

Bold values represent the statistically significant difference between median values after false discovery rate (FDR) correction for multiple comparisons over 33 cortex regions. FreeSurfer was used for cortical reconstruction and volumetric segmentation. The results of the region of interest (ROI) analyses are shown as raw and FDR corrected *p*-values.

## 4. Discussion

To our knowledge, this is the first study to evaluate the effect of maternal smoking during pregnancy on brain volumes in adolescents born very preterm. Our results showed no differences in the absolute brain volumes between exposed and unexposed group at 13 years. However, smoking exposure was associated with smaller gray matter ICV normalized volumes in the inferotemporal and parahippocampal regions, and with smaller surface area ICV normalized volume in the parahippocampal and postcentral regions.

Regarding the same very preterm cohort as in the present study, smoking exposure has been shown to associate with smaller frontal lobe and cerebellar volumes, but not with total brain volumes, in brain MRI at term (Ekblad et al., 2010). Further, full-term infants exposed to maternal smoking during pregnancy have been reported to have smaller head circumference at birth, reflecting smaller total brain volumes compared to unexposed infants (Jaddoe et al., 2007; Ekblad et al., 2015). A recent prospective population-based cohort study showed that exposure to smoking throughout pregnancy was associated with more global effects of smoking exposure compared to our present findings, such that exposed children born full-term had for example, smaller total brain volumes compared to unexposed at the age of 10 years (Zou et al., 2022).

Fetal head growth has been shown to lag by 0.13 mm per week among fetuses of smoking mothers compared to fetuses of non-smokers measured by repeated ultrasound examinations during mid- and late pregnancy (Roza et al., 2007). In our study population, prenatal exposure to smoking ended at the birth at mean gestational age of 29 weeks. Thus, the duration of smoking exposure has been significantly shorter among our study population compared to full-term infants (gestational age ≥ 37 weeks) in previous studies (El Marroun et al., 2014; Zou et al., 2022). Very preterm birth might have, in turn, somewhat protected our study population from the negative effects of continued smoking exposure on brain development, as smoking was not associated with smaller total brain volumes in adolescents born very preterm. Based on previous studies, it seems that smoking cessation in the early stages of pregnancy allows thereafter undisturbed head growth and development of the fetal brain (Roza et al., 2007; Zou et al., 2022). Zou et al. (2022) found that maternal smoking exposure only during the first trimester of pregnancy showed no differences in brain morphology compared with unexposed children at 10 years of age.

The regional volumetric changes observed in adolescent prenatal smoking-exposed brains in the current study differ from those previously reported in the same very preterm population at term (Ekblad et al., 2010). This is most likely explained by the differences in MRI equipment and image post-processing methods providing more sophisticated information. In our prior study, the ROIs were manually delineated only for six regions at term, whereas in this current study at 13 years, cortical parcellations and volume segmentations were automated. We found that smoking was associated with smaller gray matter and surface area volume in the parahippocampal region. Interestingly, animal studies have provided compelling evidence that nicotine exposure reduces the cell number and size of the hippocampus, midbrain, and cerebral cortex and alters cholinergic function of the hippocampus (England et al., 2015; Gavini et al., 2021). More research is needed to study what is the clinical significance of the volumetric findings of the present study.

In infants born full-term, both volumetric and functional alterations of the brain, as well as increased risk for behavioral problems in adolescence have been observed after maternal smoking exposure during pregnancy (Ekblad et al., 2010, 2020; Bublitz and Stroud, 2012; El Marroun et al., 2014; Zou et al., 2022). In very preterm infants, maternal smoking during pregnancy has been found to increase the risk for suboptimal cognitive and neuropsychological development up to 5 years of age (Ylijoki et al., 2019). There is need for future studies examining the effects of smoking and nicotine exposure on brain development as well as later neurobehavioral functioning in adolescents born very preterm.

### 4.1. Strengths and limitations

A major strength of our study was the prospective study design with a long follow-up time of 13 years. There were no clinically significant differences in perinatal background characteristics or maternal smoking status during pregnancy between study participants and drop-outs strengthening the reliability of the results. Even though only adolescents born very preterm in 2004–2006 were included because of the changed imaging equipment used at term, the rate of maternal smoking exposure was similar (21% vs. 18%) compared with the whole PIPARI Study cohort, including also participants born between 2001–2003. Another strength of this study was that a sophisticated MRI scanner and imaging sequences were used. Image post-processing was performed with FreeSurfer which is regarded as the method of choice in brain volumetric analyses.

Our study has several limitations. Information on maternal smoking was dichotomous, and we did not have data on possible smoking cessation during pregnancy. However, according to the report Finnish Institute for Health and Welfare (2020), only 22% of women stopped smoking during pregnancy during the study years compared to the current cessation rates of 56%. Unfortunately, we did not have information on partners’ smoking status available nor did we use any biomarkers to verify maternal smoking status. The prevalence of smoking in our study population was at the same level as generally in Finland at that time (the report Finnish Institute for Health and Welfare, 2020), thus the information on smoking status in our study seems reliable. We lacked reliable information on the number of smoked cigarettes per day, although, it has been shown that even a small number of smoked cigarettes affects fetal head growth during pregnancy (Jaddoe et al., 2007). Full-term controls would have enabled clarification on whether our findings are associated only with very preterm birth, which limits the generalizability of the results. Even though all adolescents exposed to maternal smoking during pregnancy in the present study were boys, the limited number of these adolescents did not enable multifactorial statistical analysis. Therefore, future studies with larger data are needed to evaluate the effect of possible gender bias regarding maternal smoking during pregnancy. There is also need for further studies with larger data and neurological clinical measures on, e.g., emotion, memory, or motion abilities. Another limitation is the lack of adjustment for puberty status of the adolescents. Prenatal smoking exposure might lead to 1–3 months earlier onset of puberty according to a recent study (Brix et al., 2019), which is unlikely to affect the results of this study.

We did not have information on maternal use of other nicotine containing products than cigarette smoking during pregnancy. At the time of the recruitment of our study population, smoking was the most significant source of nicotine exposure. Since then, the use of nicotine replacement therapy during pregnancy has increased, and other sources of exogenous nicotine, e.g., e-cigarettes and nicotine pouches have entered the market. Nicotine is a very harmful substance, neurotoxic to the fetus, thus the use of nicotine during pregnancy should be avoided (McGrath-Morrow et al., 2020). In the future, there is a need for studies on the effects of pure nicotine on fetal brain development.

In conclusion, although no differences were found in the absolute volumes, maternal smoking exposure during pregnancy was associated with smaller gray matter volumes proportional to intracranial volume in the inferotemporal and parahippocampal regions, and smaller surface area volume in the parahippocampal and postcentral regions. The results of our study imply that maternal smoking exposure during pregnancy have long-term effects on brain volumes up to 13 years in adolescents born very preterm. Our findings emphasize the importance of smoking-free pregnancy.

## Data availability statement

The original contributions presented in this study are included in the article/Supplementary material, further inquiries can be directed to the corresponding author.

## Ethics statement

The studies involving human participants were reviewed and approved by the Ethics Review Committee of the Hospital District of Southwest Finland. Written informed consent to participate in this study was provided by the participants’ legal guardian/next of kin.

## The PIPARI study group

Mikael Ekblad; Satu Ekblad RN; Eeva Ekholm; Linda Grönroos; Leena Haataja; Mira Huhtala; Jere Jaakkola; Eveliina Joensuu; Riikka Korja; Katri Lahti; Helena Lapinleimu; Liisa Lehtonen; Tuomo Lehtonen; Marika Leppänen; Annika Lind; Petriina Munck; Anna Nyman; Riitta Parkkola; Liisi Ripatti; Päivi Rautava; Katriina Saarinen; Tiina Saarinen; Virva Saunavaara; Sirkku Setänen; Matti Sillanpää; Suvi Stolt; Päivi Tuomikoski RN; Karoliina Uusitalo; and Milla Ylijoki.

## Author contributions

PN and HM contributed to the data curation and post-processing of MRI data. PN, HM, and SS contributed to the formal analysis. RP contributed to the funding acquisition. PN, HM, VS, and RP contributed to the methodology. RP and SS contributed to the project administration. ME, PN, HM, and SS contributed to the writing—original draft. All authors contributed to the article, conceptualization, investigation, writing—review and editing, and approved the submitted version.
