# Supplementary material for: Maternal smoking during pregnancy negatively affects brain volumes proportional to intracranial volume in adolescents born very preterm
Source: Front Hum Neurosci. 2023 Jan 5;16:1085986. doi: 10.3389/fnhum.2022.1085986 (PMC9849910; doi:10.3389/fnhum.2022.1085986)
Supplement: Supplementary file 1 [file Table_1.DOCX]

Supplementary Material

# Supplementary Tables

**SUPPLEMENTARY TABLE 1** Regions of interests (ROI) for volumetric analyses of adolescents born very preterm exposed and unexposed to maternal smoking during pregnancy, as depict in Freesurfer that was used to get the volumetric estimates.

| **Subcortical Regions** | **Cortical Parcellations** | |
| --- | --- | --- |
| Left-Cerebellum-White-Matter | caudalanteriorcingulate | pericalcarine |
| Left-Cerebellum-Cortex | caudalmiddlefrontal | postcentral |
| Brain-Stem | cuneus | posteriorcingulate |
| Left-Hippocampus | entorhinal | precentral |
| Left-Amygdala | fusiform | precuneus |
| Left-Accumbens-area | inferiorparietal | rostralanteriorcingulate |
| Left-VentralDC | inferiortemporal | rostralmiddlefrontal |
| Right-Cerebellum-White-Matter | isthmuscingulate | superiorfrontal |
| Right-Cerebellum-Cortex | lateraloccipital | superiorparietal |
| Right-Hippocampus | lateralorbitofrontal | superiortemporal |
| Right-Amygdala | lingual | supramarginal |
| Right-Accumbens-area | medialorbitofrontal | frontalpole |
| Right-VentralDC | middletemporal | temporalpole |
| CC_Posterior | parahippocampal | transversetemporal |
| CC_Mid_Posterior | paracentral | insula |
| CC_Central | parsopercularis |  |
| CC_Mid_Anterior | parsorbitalis |  |
| CC_Anterior | parstriangularis |  |

**SUPPLEMENTARY TABLE 2** Differences in subcortical region matter volumes between adolescents born very preterm exposed and unexposed to maternal smoking during pregnancy. Freesurfer was used for volumetric segmentation. The results of the ROI analyses are shown as raw and FDR corrected p-values.

| **ROI** | **p-value** | **FDR p-value** |
| --- | --- | --- |
| Left-Cerebellum-White-Matter | 0.987 | 1.000 |
| Left-Cerebellum-Cortex | 0.528 | 0.707 |
| Brain-Stem | 0.222 | 0.666 |
| Left-Hippocampus | 0.146 | 0.655 |
| Left-Amygdala | 0.467 | 0.707 |
| Left-Accumbens-area | 0.262 | 0.671 |
| Left-VentralDC | 0.711 | 0.799 |
| Right-Cerebellum-White-Matter | 1.000 | 1.000 |
| Right-Cerebellum-Cortex | 0.339 | 0.671 |
| Right-Hippocampus | 0.137 | 0.655 |
| Right-Amygdala | 0.594 | 0.713 |
| Right-Accumbens-area | 0.528 | 0.707 |
| Right-VentralDC | 0.373 | 0.671 |
| CC_Posterior | 0.183 | 0.658 |
| CC_Mid_Posterior | 0.146 | 0.655 |
| CC_Central | 0.322 | 0.671 |
| CC_Mid_Anterior | 0.049 | 0.655 |
| CC_Anterior | 0.550 | 0.707 |
